# “Non-Toxic” Proteins of the Botulinum Toxin Complex Exert *In-vivo* Toxicity

**DOI:** 10.1038/srep31043

**Published:** 2016-08-10

**Authors:** Shin-Ichiro Miyashita, Yoshimasa Sagane, Tomonori Suzuki, Takashi Matsumoto, Koichi Niwa, Toshihiro Watanabe

**Affiliations:** 1 Department of Food and Cosmetic Science, Faculty of Bioindustry, Tokyo University of Agriculture, 196 Yasaka, Abashiri 099-2493, Japan; 2 Department of Nutritional Science and Food Safety, Faculty of Applied Bioscience, Tokyo University of Agriculture, 1-1-1 Sakuragaoka, Setagaya-ku, Tokyo 156-8502, Japan; 3 Rigaku Corporation, 3-9-12 Matsubara-Cho, Akishima 196-8666, Japan

## Abstract

The botulinum neurotoxin (BoNT) causes muscle paralysis and is the most potent toxin in nature. BoNT is associated with a complex of auxiliary “Non-Toxic” proteins, which constitute a large-sized toxin complex (L-TC). However, here we report that the “Non-Toxic” complex of serotype D botulinum L-TC, when administered to rats, exerts *in-vivo* toxicity on small-intestinal villi. Moreover, Serotype C and D of the “Non-Toxic” complex, but not BoNT, induced vacuole-formation in a rat intestinal epithelial cell line (IEC-6), resulting in cell death. Our results suggest that the vacuole was formed in a manner distinct from the mechanism by which *Helicobacter pylori* vacuolating toxin (VacA) and *Vibrio cholerae* haemolysin induce vacuolation. We therefore hypothesise that the serotype C and D botulinum toxin complex is a functional hybrid of the neurotoxin and vacuolating toxin (VT) which arose from horizontal gene transfer from an ancestral BoNT-producing bacterium to a hypothetical VT-producing bacterium.

The botulinum neurotoxin (BoNT), the most potent toxin in nature, is categorised into seven distinct serotypes, A through G. Serotypes A, B, E, and F are strongly associated with human botulism, whereas serotypes C and D cause animal and avian botulism. BoNT consists of three domains, each of which makes up a third of the protein: the C-terminal third facilitates binding to nerve cells via dual receptor binding, while the central third facilitates the translocation of the toxin into the cytosol. The N-terminal third possesses zinc-dependent metalloprotease activity that cleaves the specific protein involved in neurotransmitter release, resulting in the muscular paralysis caused by BoNT^1^.

BoNT in *Clostridium botulinum* spontaneously associates with nontoxic nonhaemagglutinin (NTNHA), yielding the BoNT/NTNHA complex (M-TC). Furthermore, in strains of serotypes A–D, three types of haemagglutinins (HAs; three HA-70, three HA-17, and six HA-33) are associated with the M-TC, resulting in formation of the 14-mer large-sized toxin complex (L-TC)^2^,^3^ (Fig. 1a). BoNT and the “Non-Toxic” complex dissociate from the L-TC in a pH-dependent manner. Orally ingested BoNT encounters several barriers before entering the human or animal body. Harsh digestive conditions in the gastrointestinal tract represent the first of these barriers. While the BoNT protein in complex with the NTNHA protein exhibits notable tolerance to these digestive conditions^4^, the BoNT protein when it is free of the non-toxic proteins is easily degraded into small fragments in the stomach and intestine. The NTNHA protein thus plays a role in protecting BoNT against the digestive conditions in the gastrointestinal tracts of animals and humans. The second barrier is the intestinal wall. In the absence of the non-toxic protein, BoNT can be transported through the intestinal epithelial cells. The HA proteins furthermore facilitates the trans-epithelial absorption of the toxin complex via nine glycan-binding sites on the HA-33 and HA-70 proteins^5^,^6^. It has also been shown that the serotype A BoNT complex disrupts E-cadherin adhesion, thereby disrupting interactions between epithelial cells^7^. This process may facilitate the trans-epithelial transport of the toxin complex.

A number of recent studies have served to gradually reveal the physical role of the “Non-Toxic” complex in the botulinum toxin complex. In the current study, we demonstrate that the “Non-Toxic” complex of serotype D botulinum L-TC, when administered to rats, exerts *in-vivo* toxicity on small-intestinal villi.

## Results

### Toxicity found in the serotype C and D botulinum “Non-Toxic” complex

**Figure 1:**
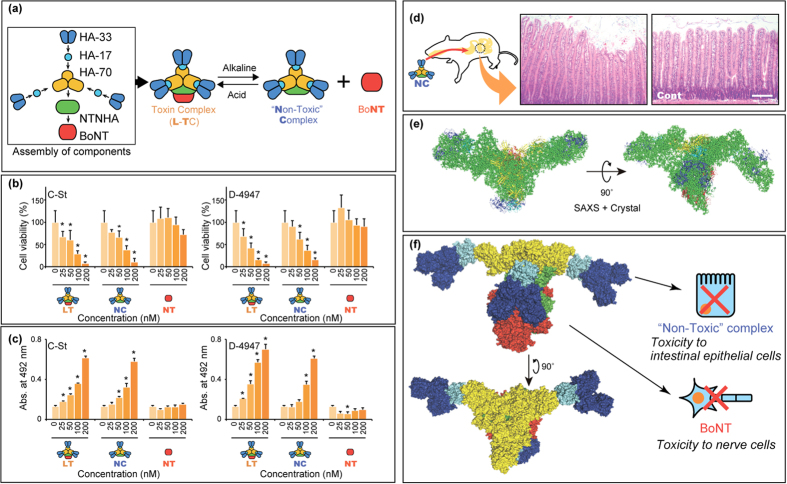
Cytotoxicity found in the “Non-Toxic” complex of serotype D botulinum toxin complex. (**a**) Formation of the toxin complex and its subsequent association/dissociation process. The BoNT associates with the NTNHA and HA-70, HA-17, and HA-33 and forms a toxin complex, L-TC. Under alkaline conditions, L-TC dissociates into the “Non-Toxic” complex and BoNT. Under acidic conditions, the “Non-Toxic” complex and BoNT form a complex. (**b**) Viability of cells exposed to the “Non-Toxic” complex (NC), L-TC (LT), and BoNT (NT). Cell viabilities relative to those of untreated control cells (0 nM) were calculated and are reported as percentages. (**c**) Cytotoxicity of the “Non-Toxic” complex, L-TC, and BoNT assessed by an LDH leakage assay. Experiments for panel b,c were performed thrice and in triplicate, with representative results shown as mean ± SD. Asterisks denote significant differences (*p* < 0.05) in viability or leakage levels between toxin-treated and untreated cells. (**d**) *In vivo* toxic effect of the “Non-Toxic” complex on rat intestinal cells. Rats were orally administered PBS (right panel, Cont) or “Non-Toxic” complex (left panel). Scale bars: 200 μm. (**e**) Solution structure of the L-TC resembling a bird spreading its wings as revealed by small-angled X-ray scattering (SAXS). The crystal structure of serotype A BoNT/NTNHA complex (red for BoNT and light green for NTNHA), serotype C HA-70 trimer (yellow), and serotype D HA-33/HA-17 trimer (blue for HA-33 and light blue for HA-17) were docked into the SAXS dummy residual model. (**f**) Proposed solution structure of the serotype D L-TC and hybrid toxin model of the BoNT and “Non-Toxic” complex. Findings are shown in the panels.

The cytotoxicities of the L-TC, BoNT, and “Non-Toxic” protein complex from the L-TC, produced by the serotype C and D strains, were examined in the IEC-6 rat intestinal epithelial cell line. The L-TC, BoNT, and “Non-Toxic” protein complex preparations used in this investigation were purified to a high degree from a culture of *C. botulinum* serotype C strain Stockholm (C-St) and D strain 4947 (D-4947) (Supplementary Fig. 1). As shown in Fig. 1b, the number of viable cells decreased in a dose-dependent manner when exposed to the L-TC and “Non-Toxic” complex. Leakage of cellular lactate dehydrogenase (LDH), moreover, increased upon the addition of the L-TC and “Non-Toxic” complex (Fig. 1c); while BoNT did not induce a decrease in the number of viable cell or increase LDH leakage (Fig. 1b,c, respectively). These results suggest that the “Non-Toxic” complex of the serotype C and D L-TC, but not BoNT, induce cell death in IEC-6 cells. Components of the serotype C and D “Non-Toxic” complexes share a high level of amino acid sequence similarity (87–97% identity). The serotype D complex was therefore used in subsequent experiments. To determine whether the “Non-Toxic” complex has cytotoxic effects on intestinal cells *in vivo*, rats were furthermore orally treated with the “Non-Toxic” complex. After 36 h, jejunum tissue sections were prepared from the rats. In “Non-Toxic” complex-treated rats, jejunal epithelial cells were detached and exhibited marked destruction of the mucosal surface in the upper regions of villi (Fig. 1d), implying that the “Non-Toxic” complex has cytotoxic effects on cells *in vivo*.

### Three-dimensional structure of the botulinum toxin complex

The conformational structure of the botulinum toxin complex has been revealed to some extent by transmission electron microscopic (TEM) analyses^3^,^8^ and crystal structure analysis of subcomplexes such as the M-TC^9^ and HA complex^10^. Here, the solution structure of the whole serotype D L-TC was assessed using small-angle X-ray scattering (SAXS) to study the functionally active structure. Figure 1e shows the SAXS image for the L-TC, which resembles the shape of a bird spreading its wings. The L-TC was previously reported^2^ to have three “arms” of HA-33/HA-17 trimers (Fig. 1a); however, the SAXS image here reveals only two “arms”. To construct a solution structure model of L-TC, crystal structures for a single molecule of serotype A M-TC^9^, a single-molecule of serotype C HA-70 trimer^10^, and three molecules of serotype D HA-33/HA-17 trimers^3^ were manually superimposed into the SAXS image, such that all molecules were placed into the image (Supplemental Movie 1). In this model, the M-TC can be superimposed into the portion that corresponds to the “body” of the bird shape. Three HA-33/HA-17 molecules are associated with the M-TC via the HA-70 trimer. Moreover, two of the three HA-33/HA-17 molecules correspond to the “wing” of the bird in this model, and another one binds to both the HA-70 trimer and BoNT in M-TC via HA-17/HA-70 and HA-33/BoNT interactions. Sharma and Singh (2004) reported that the HA-33 molecule interacts with BoNT via a strong protein-protein interaction^11^, which is consistent with a hypothetical HA-33/BoNT interaction proposed in our model of the botulinum L-TC. In this model, the serotype D botulinum L-TC (in addition to the serotype C toxin) is composed of a neurotoxic BoNT and a “Non-Toxic” complex exhibiting vacuolating cytotoxicity via an interaction between NTNHA/HA-70 and BoNT/HA-33 (Fig. 1f). In our model for the solution structure of L-TC, the HA-70 trimer appears more accessible to cells compared with in the three-arm model for the TEM image of the L-TC. Recently Amatsu *et al*. reported the crystal structure of the serotype C whole HA complex^10^. In the centre of the structure, three HA-70 molecules form a central pore with a diameter of ~26 Å—the same size as the pore formed by the pore-forming toxin hemolysin produced by *Staphylococcus aureus*^12^. The SAXS image therefore indicates that the central pore may indeed access the cell membrane when the “Non-Toxic” complex binds to cells.

### “Non-Toxic” complex induces cell morphology changes and vacuolation

**Figure 2:**
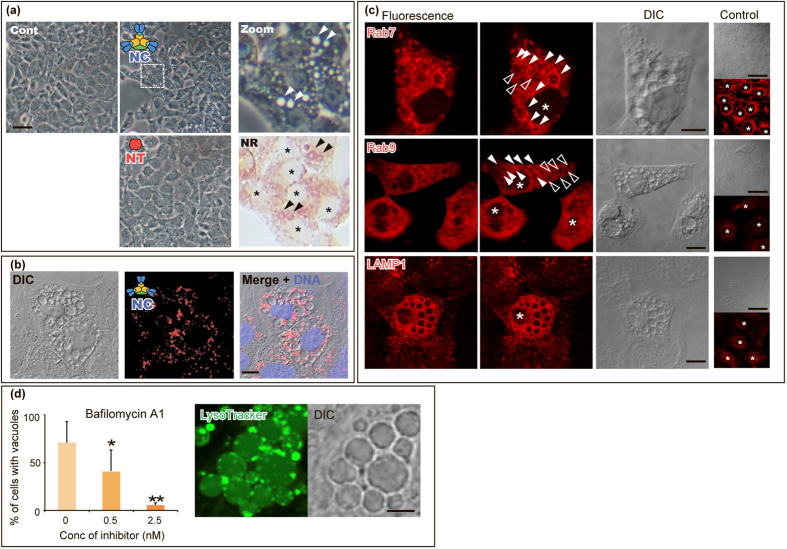
“Non-Toxic” complex induced vacuoles in IEC-6 cells. (**a**) Vacuoles induced by exposure to the “Non-Toxic” complex. Vacuole formation was induced when the IEC-6 cells were exposed to the “Non-Toxic” complex (NC; 50 nM for 24 h); however, no vacuole formation was observed in cells exposed to BoNT (NT; 50 nM for 24 h). Control (Cont) samples were cells assessed prior to exposure. Higher magnification (5×) is shown for the region indicated by a dashed square. The vacuoles induced by the “Non-Toxic” complex were peripherally stained with neutral red (NR). Asterisks indicate nuclei of the cells. Scale bar: 50 μm. (**b**) The “Non-Toxic” complex surrounds the vacuoles. The cells were exposed to the Cy5-labelled “Non-Toxic” complex (red) and were analysed by confocal microscopy. Chromosomal DNA was stained with DAPI (blue). Scale bar: 20 μm. (**c**) “Non-Toxic” complex-induced vacuoles were co-localised with late-endosome- and lysosome-specific proteins. Post induction of the vacuoles by exposure to the “Non-Toxic” complex, the Rab7, Rab9, and LAMP1 proteins were immunostained with antibodies. The control shows the localisation of each protein in cells that were not exposed to the “Non-Toxic” complex. Scale bars: 20 μm for vacuole-induced cells and 50 μm for control cells. (**d**) Involvement of the endosomal proton pump on “Non-Toxic” complex-induced vacuolation. The endosomal proton pump inhibitor—bafilomycin A1—inhibited “Non-Toxic” complex-induced vacuolation (left panel) in a dose-dependent manner. Experiments were performed thrice and in triplicate; with representative results reported as mean ± SD. Asterisks denote significant differences in the number of the cells with vacuoles (*p < 0.05, **p < 0.01) compared with those in the control in the presence of inhibitor solvent, 0.08% ethanol, alone (corresponding to the 25 nM). LysoTraker indicates the peripheral acidification of vacuoles induced by the “Non-Toxic” complex (middle and left panels). Scale bar: 5 μm.

Interestingly, when IEC-6 cells were exposed to the “Non-Toxic” complex, vacuole-formation was induced in the cytoplasm (Fig. 2a), while vacuole-formation was not induced in cells treated with BoNT. The neutral red (NR) stain stained peripheral regions of the induced vacuoles, whereas vacuoles induced by *Helicobacter pylori* vacuolating toxin (VacA) were completely stained with the NR^13^. As shown in Fig. 2b, the “Non-Toxic” complexes surrounded the vacuoles in the vacuole-induced cells, rather than entering the vacuoles, suggesting that the vacuoles were not produced to degrade the invaded “Non-Toxic” complex through autophagy.

### Characterisation of “Non-Toxic” complex-induced vacuoles

Next, to understand the origin of the vacuoles induced by the “Non-Toxic” complex, the Rab GTPases and marker proteins in cells with “Non-Toxic” complex-induced vacuoles were immunostained (Fig. 2c and Supplementary Fig. 2; Summarised in Table 1). Immunostaining with anti-Rab7 yielded fluorescence around the “Non-Toxic” complex-induced vacuoles; however, not all the vacuoles co-localised with Rab7, which is known to play a role in controlling aggregation of late endosomes and lysosomes. Upon anti-Rab9 staining, some vacuoles exhibited fluorescence. The Rab7 and Rab9 GTPases are specific to late endosomes and have different biological functions. Immunostaining furthermore demonstrated that all vacuoles were surrounded by the lysosomal-associated membrane protein 1 (LAMP1), which is associated with late endosomes and lysosomes. No associations, however, were observed between the “Non-Toxic” complex-induced vacuoles and various other marker proteins: early endosome-specific EEA-1 and Rab5, recycling endosome-specific Rab11, Golgi-specific GM130, and endoplasmic reticulum-specific calnexin. The immunostaining results thus demonstrate that the “Non-Toxic” complex selectively associates with late endosomal and lysosomal compartments, resulting in the formation of vacuoles. In contrast to the botulinum “Non-Toxic” complex shown in this study to be associated with both Rab7- and Rab9-associated compartments, other vacuolating toxins VacA (*H. pylori*)^14^ and CARDS toxin (*M. pneumoniae*)^15^ were found to be specifically associated with only the Rab7- or Rab9-associated compartments, respectively.

## Discussion

**Figure 3:**
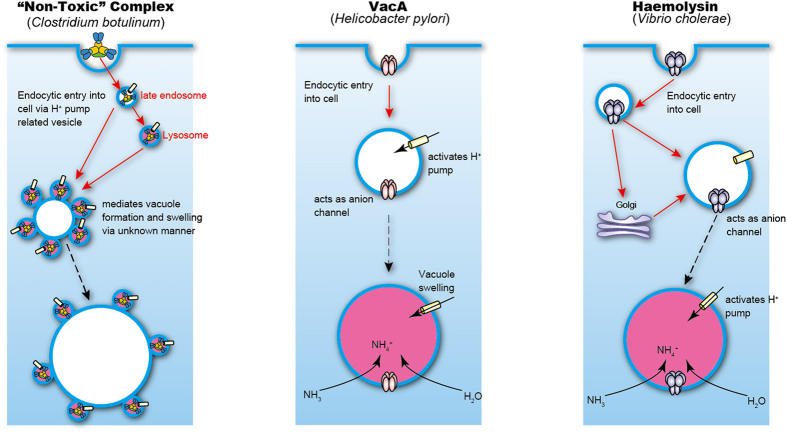
Working model for the vacuolation mechanism of the botulinum “Non-Toxic” complex in comparison to the vacuolation mechanisms of other vacuolating toxins. The left panel illustrates the working model of the botulinum “Non-Toxic” complex-induced vacuolating mechanism. The “Non-Toxic” complex enters into the cell in a sialic acid-dependent receptor-mediated manner. The process appears to involve proton pump-related vesicle formation. The “Non-Toxic” complex-containing vesicles generated from late endosomes and lysosomes then mediate the formation and swelling of vacuoles via an unknown mechanism. Central and right panels illustrate the mechanisms of action of VacA^17^ and *V. cholerae* haemolysin^34^. The VacA toxin is inserted into the lipid bilayer of cells and forms an oligomer, which then acts as an anion channel and activates the proton pump. The *V. cholerae* haemolysin also forms oligomers on the cell membrane. The oligomeric haemolysin forms larger and less specific channels than VacA. Entrance of ions into these channels induces osmotic swelling of the vacuoles in both VacA- and haemolysin-treated cells.

Figure 3 illustrates a working model for the “Non-Toxic” complex-induced vacuolation mechanism, as well as a comparison with other toxins (*H. pylori* VacA and *V. cholerae* haemolysin) which have been shown to induce cell vacuolation. Serotype C and D botulinum L-TC is internalised into intestinal cells. Nishikawa *et al*. reported that pre-incubation of HT-29 cells with the O-glycosylation inhibitor d,l-threo-1-phenyl-2-hexa-decanoylamino-3-morpholino-propanol reduces serotype C L-TC internalisation^16^, suggesting that L-TC may be taken up by cells with an O-linked oligosaccharide derived from glycoproteins, similar to mucin on the cell surface. It was, moreover, demonstrated that the serotype C L-TC binds to mucin derived from the bovine submaxillary gland via sialyl oligosaccharide. The internalisation of L-TC is independent of the presence of BoNT and occurs via binding of HA-33 to a hypothetical receptor on the cell surface. Therefore, in the first step of the vacuolating process, the botulinum “Non-Toxic” complex is internalised into cells via receptor-mediated endocytosis.

VacA^17^ and *V. cholerae* haemolysin^18^ are incorporated into lipid bilayers of vesicle membranes, forming polymeric pores and thus acting as channels for anions (central and right panels, Fig. 3). The anions then act as counter ions for the protons that enter into the cells through the proton pump. This process results in osmotic swelling of vacuoles, and the toxin-induced vacuoles thus become acidified. The crystal structure of the serotype C whole HA complex reveals the central pore formed by the trimeric HA-70 proteins^10^, and the SAXS data reported here indicate that the cell surface is accessible to the central pore. The depth of this pore is, however, insufficient to form a channel on the lipid bilayer of the vesicle and the “Non-Toxic” complex is thus not likely to act as an ion channel. The peripheral regions of the toxin-induced vacuoles were stained with NR; however, stain was not incorporated into the vacuoles, which suggests that the large botulinum “Non-Toxic” complex-induced vacuoles are surrounded by acidified small vesicles. The LysoTracker signals observed also support this model of acidified vesicles surrounding the vacuoles. The induction of vacuole formation by the botulinum “Non-Toxic” complex thus seems to differ from the vacuole induction observed for VacA and *V. cholerae* haemolysin. The internalised botulinum “Non-Toxic” complex is likely to associate with the late endosome and lysosome, since the induced vacuoles or surrounding vesicles were co-localised with specific proteins for these compartments.

As in the cases of vacuole formation mediated by the VacA^19^, *V. cholerae* haemolysin^18^, CARDS toxin^15^, and EHEC-Vac^19^, vacuole formation induced by the botulinum “Non-Toxic” complex was inhibited by bafilomycin A1—a vacuolar-type proton pump inhibitor. Since the “Non-Toxic” complex-induced vacuoles in the cells were partially acidified, however, the vacuole formation observed in this study differs from vacuolation induced by other toxins. Previous reports indicate that bafilomycin A1 inhibits receptor-mediated endocytosis in the rat sinusoidal endothelial cells^20^,^21^ and hepatocytes^22^, indicating that the vascular-type proton pump is essential for the acidification of the endocytic compartment and for the pH gradient between the compartments and the cytoplasm, which in turn is required for receptor-mediated endocytosis. Inhibition of “Non-Toxic” complex-induced vacuolation by bafilomycin A1 may thus result from disruption of endocytosis rather than from the prevention of vacuole swelling by proton pumping. The mechanism of the vacuole swelling observed in this study remains unclear: the acidified vesicles are generated from late endosomes and lysosomes, including “Non-Toxic” complex-induced vacuoles, in an unknown manner.

**Figure 4:**
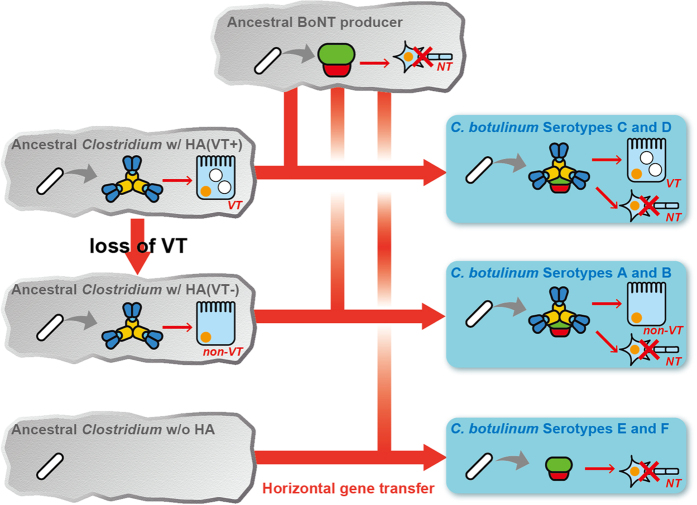
Model of horizontal BoNT gene transfer of the hybrid botulinum toxin complex. Here, the ancestral BoNT gene cluster is transferred into the clostridial bacterium producing an HA complex with vacuolating toxicity [HA (VT^+^)], yielding an ancestor of the *C. botulinum* serotype C and D strains. Meanwhile, the serotype A and B strains also produce the toxin complex with HA components, but these strains do not exhibit vacuolating cytotoxicity. Therefore, the ancestral BoNT gene cluster transfers into the HA complex-producing bacterium [HA (VT^−^)], whose vacuolating toxicity is lost during its molecular evolution. The BoNT gene cluster transfer into a clostridial bacterium with no HA gene may yield ancestors of the serotype E and F strain.

From the findings reported here, we hypothesise that the serotype C and D botulinum L-TC is a functional hybrid that exhibits both neurotoxicity and vacuolating cytotoxicity resulting from the horizontal transfer of the BoNT-NTNHA gene cluster into a hypothetical VT-producing bacterium (Fig. 4). We thus propose that the VT is an ancestor of current HA proteins. *C. botulinum* is taxonomically defined as a species based on only one phenotype, i.e., the production of the BoNT, but is genetically related to various other *Clostridium* species^23^. Some *C. botulinum* species furthermore contain multiple BoNT genes with different serotypes^24^. Various *Clostridium* species produce BoNT and some strains express combinations of BoNT genes, strongly suggesting that the BoNT gene was derived from a common ancestor and was horizontally transferred between *Clostridium* species. Among bacteria, the Clostridia group produces the largest number of toxins. Interestingly, the related toxin genes have been identified between distinct *Clostridium* species, indicating horizontal gene transfers of not only BoNT-related genes between strains^23^. Doxey *et al*. demonstrated that the BoNT and NTNHA proteins evolved from a common collagenase-like ancestor via gene duplication^25^. We recently showed that at least three types of HA-33 genes exist, and that these genes are shuffled among the serotype C and D strains independently of the BoNT serotype^26^. The duplication, reshuffling, and rearrangement of the genes in the BoNT gene cluster and its neighbour genes may have led to the functional hybridisation resulting in the botulinum toxin complex. Recently, Mansfield *et al*.^27^ furthermore identified the gene cluster encoding the BoNT and NTNHA homologs in a non-*Clostridium* species, *Weissella oryzae* SG25, which contains no HA genes. These findings may indicate that the horizontal transfer of the BoNT gene always accompanies the NTNHA gene, and that the BoNT/NTNHA complex (M-TC) in serotypes A–D forms the HA complex by chance during the evolution of the bacterium. The serotype E and F strains, however, do not contain the genes for HAs. Instead of the HA gene cluster, these strains possesses the *orfx* gene cluster, the function of which is unclear^28^. During the horizontal gene transfer event, the serotype E and F BoNT/NTNHA gene cluster appears to have “jumped” upstream of the *orfx* gene cluster, whereas the corresponding clusters of other serotypes were inserted downstream of the HA gene cluster.

Recently, Sugawara *et al*.^29^ demonstrated that the serotype C HA complex, but not the serotype B HA complex, affects cell viability of MDCK-I cells. The vacuolating cytotoxicity therefore seems to be restricted to serotypes C and D. Barriers represented by the intestinal wall must be overcome in order for orally ingested BoNT to enter an animal or human body. Free from the “Non-Toxic” complex, BoNT can be transported across the intestinal epithelial cell layer; however, the formation of L-TC with the “Non-Toxic” complex significantly enhances toxin transport through the cell layer^5^. BoNT may thus have evolved to overcome the intestinal wall barrier via complex formation with VT, a hypothetical ancestor of the botulinum “Non-Toxic” complex or HA complex, through a horizontal gene transfer event (serotypes C and D). Serotype B (in addition to A) L-TC, however, binds directly to E-cadherin and disrupts E-cadherin-mediated cell-cell interactions^30^. The “Non-Toxic” complex of serotypes A and B may therefore have lost its vacuolating cytotoxicity effects before or after the horizontal transfer of the BoNT gene, and instead evolved to disrupt E-cadherin-mediated cell-cell interactions.

In this study, the novel cytotoxicity of the “Non-Toxic” complex of the botulinum toxin complex was identified. The botulinum toxin complex was hypothesised to be a hybrid of the neurotoxin and “Non-Toxic” cytotoxic protein complex produced by an ancient gene transfer event from the neurotoxin-producing bacterium to the vacuolating toxin-producing bacterium. The “Non-Toxic” complex thus seems to enable the toxin complex to effectively overcome the intestinal barrier against toxin traffic. This hypothesis is supported by the fact that in botulism disease in livestock, animals sometimes exhibit haemorrhage in their intestines and vessels^31^,^32^. These symptoms have been thought to be due to enterotoxin concomitantly produced by the serotype C and D *C. botulinum* strains; however, our findings indicate that the botulinum “Non-Toxic” proteins may also play a role in these symptoms. Further investigation into the cytotoxicity of the “Non-Toxic” complex is required for a greater understanding of this research area to be gained, and such investigations may contribute toward the development of strategies in the prevention of botulism disease. An in-depth knowledge of the toxin traffic mechanisms of the “Non-Toxic” complex would furthermore allow for the development of novel oral drug-delivery systems in which peptide and protein pharmaceuticals are attached to the botulinum toxin complex instead of the BoNT protein and in which the “Non-Toxic” complex is modified to exhibit only moderate cytotoxicity.

## Methods

### Production and purification of the botulinum toxin complex and non-toxic component

The production of botulinum toxin complexes as well as the separation of the “Non-Toxic” complex and BoNT from the toxin complexes of *C. botulinum* serotype D strain 4947 and serotype C strain Stockholm were performed as described previously^33^.

### Small-angled X-ray scattering

Small-angle X-ray scattering (SAXS) measurement of the L-TC (5 mg/mL) in 50 mM acetate buffer (pH 4.0) was performed using a Rigaku BioSAXS-1000 and a 10–20 μL protein solution. In total, eight datasets were collected after 120 min exposure (15 min per dataset) and raw data were analysed using the SAXSLab software package (Rigaku). SAXS curves were generated after subtracting the scattering due to the solvent in the protein solution using the PRIMUS program from the AT-SAS package.

### Cell culture

The rat small intestinal epithelial cell line IEC-6 was purchased from the RIKEN BioResource Center (Tsukuba, Japan). Cells were grown in Dulbecco’s modified Eagle medium (DMEM) supplemented with 10% foetal bovine serum (FBS), penicillin (100 IU mL^−1^), and streptomycin (100 μg mL^−1^). Cells were maintained in a humidified environment of 5% CO_2_ and 95% air at 37 °C. Culture medium was replaced every 2–3 days.

### Cytotoxicity and cell viability assays

Cytotoxicity was assessed using a cytotoxicity detection kit (LDH; Roche). IEC-6 cells were cultured in 96-well culture plates (Corning) at 25% confluence. After 1 day of culture, cells were washed with DMEM containing 10% FBS and incubated in 100 μL of medium containing test samples for 24 h. Measurement of cytotoxicity was performed according to the manufacturer’s instructions. Absorbance was measured at 492 nm with a reference at 600 nm and cell viability was determined using the Cell Counting Kit-8 (CCK-8, Dojindo, Tokyo, Japan), where absorbance was measured at 450 nm.

### Neutral red assay

Cells were incubated with 50 nM “Non-Toxic” complex at 37 °C for 24 h to induce vacuolation. To quantify the extent of vacuolation, cells in 96-well culture plates were incubated for 6 min at room temperature with 50 μL freshly prepared 0.05% NR in PBS containing 0.3% bovine serum albumin (BSA). The cells were then washed three times with 200 μL PBS containing 0.3% BSA. After the addition of 100 μL ethanol (70% in water) containing 0.4% HCl, absorbance was measured at 550 nm.

### Assessing inhibition of vacuolation by bafilomycin A1

Cells were treated with various concentrations of bafilomycin A1 at 37 °C for 45 min, after which 50 nM “Non Toxic” complex was added to the cells in the presence of the bafilomycin A1. The cells were incubated for a further 6 h at 37 °C. The inhibitory effect of bafilomycin was assessed by counting the number of cells with and without vacuoles under a microscope.

### Cy5-labelled “Non-Toxic” complex-induced vacuolation

“Non-Toxic” complex was labelled with Cy5 (GE Healthcare) according to the manufacturer’s recommendations and free dye was removed by gel filtration. The extent of labelling was determined by measurement of Cy5 levels (at 650 nm) and protein concentrations (at 280 nm). Cells were seeded onto chambered glass slides (Nunc A/S, Roskilde, Denmark) and then incubated with Cy5-labelled proteins (50 nM) diluted in 10% FBS-containing DMEM for 24 h at 37 °C to allow cell vacuolation by Cy5-labelled proteins. The cells were fixed in 4% (v/v) formaldehyde (Polysciences) for 15 min before being examined under a Leica TCS laser scanning confocal microscope (Plan Apo; 40×; oil immersion; 1.25 NA objective). The resulting images were processed using dedicated Leica software (version 2.5).

### Immunofluorescent staining of vacuole-induced cells

Cells were seeded in an 8-chamber glass slide (Lab-Tek II Chambered, Nunc) at 30% confluence per chamber 18 h before treatment. The cells were then incubated in the presence or absence of 50 nM “Non-Toxic” complex for 24 h at 37 °C and were then fixed with 4% formaldehyde for 15 min at room temperature (RT). Cells were then permeabilised and blocked with PBS containing 0.3% Triton-X-100 and 5% goat serum (Wako) at RT for 1 h. Next, the cells were incubated with primary antibodies diluted to 1:200 (EEA1 and Rab5), 1:100 (Rab7, Rab9 Rab11, GM130, and Lamp1), or 1:50 (Calnexin) in PBS containing 0.3% Triton-X-100 and 1% BSA overnight at 4 °C. Secondary Alexa Fluor 647 goat anti-rabbit or anti-mouse IgG antibodies were diluted 1:500 in PBS with 0.3% Triton-X-100 and 1% BSA for 1 h at RT. Cover slips were mounted onto slides with ProLong Diamonds with DAPI (Life Science Technology) before being viewed under a laser scanning confocal microscope.

### LysoTracker assay

LysoTracker was used to stain the lysosome. Cells were seeded in an 8-chamber glass slide at 30% confluence per chamber 18 h before treatment. The cells were then incubated in the presence or absence of 50 nM “Non-Toxic” complex for 24 h at 37 °C, after which they were further incubated with 1 mM LysoTracker in DMEM lacking phenol red for 5 min at 37 °C. The living cells were examined by confocal microscopy.

### Animal experiments and histology of intestinal tissues

All animal experimentation protocols were carried out in accordance with the guidelines described in the Proper Conduct of Animal Experiments as promulgated by the Science Council of Japan and were approved by the Animal Care and Use Committee of Tokyo University of Agriculture. Male Sprague-Dawley rats (7 to 10 wk old) were used for experiments. The rats were deprived of food, but allowed free access to water. After 12 h, “Non-Toxic” complex dissolved in PBS was administered to the stomach of rats via an oral catheter (200 µg/100 g body weight). Administration was repeated after 24 h. After a further 12 h, the rats were killed and portions of jejunum located 25–30 cm from pylorus were removed. The tissues were fixed with 4% formalin and embedded in paraffin. Sections (4 μm in thickness) were cut, rehydrated, and stained in haematoxylin and eosin. The mucosa was then examined by microscopy (Zeiss Axiovert 40 CFL).

### Statistical analyses

Statistical analyses were performed using the unpaired *t*-test.

## Additional Information

**How to cite this article**: Miyashita, S.-I. *et al*. “Non-Toxic” Proteins of the Botulinum Toxin Complex Exert *In-vivo* Toxicity. *Sci. Rep.* **6**, 31043; doi: 10.1038/srep31043 (2016).

## Supplementary Material

Supplementary Information

Supplementary Movie S1

## Figures and Tables

**Table 1:** Comparison of vacuoles induced by “Non-Toxic” complex with those caused by other vacuolating toxins.

Toxin (Species)	Staining with NR	Staining with Lysotracker	Co-localisation	Effect of bafilomycin A1	Ref.
“Non-Toxic” complex (*Clostridium botulinum*)	Peripheral	Peripheral	Rab7, Rab9, Lamp1	Inhibit	This article
VacA (*Hericobacter pylori*)	Yes	Yes	Rab7, Lamp1	Inhibit	14,35,36,37
Haemolysin (*Vibrio cholerae*)	Yes	NI	Rab7, TGN46, Lamp1	Does not inhibit vacuole formation, but inhibits swelling of vacuoles	18,38,39
CARDS (*Mycoplasma pneumoniae*)	Yes	NI	Rab9, Lamp1, Lamp2	Inhibit	15
