# Supplementary material for: “Non-Toxic” Proteins of the Botulinum Toxin Complex Exert In-vivo Toxicity
Source: Sci Rep. 2016 Aug 10;6:31043. doi: 10.1038/srep31043 (PMC4978960; doi:10.1038/srep31043)
Supplement: Supplementary Information [file srep31043-s1.pdf]

“Non-Toxic” Proteins of the Botulinum Toxin Complex Exert *In-vivo* Toxicity

Shin-Ichiro Miyashita,[1] Yoshimasa Sagane [1*], Tomonori Suzuki,[2] Takashi Matsumoto,[3] Koichi Niwa,[1] and Toshihiro Watanabe[1]

[1] *Department of Food and Cosmetic Science, Faculty of Bioindustry, Tokyo University of Agriculture, 196 Yasaka, Abashiri 099-2493, Japan*

[2] *Department of Nutritional Science and Food Safety, Faculty of Applied Bioscience, Tokyo University of Agriculture, 1-1-1 Sakuragaoka, Setagaya-ku, Tokyo 156-8502, Japan*

[3] *Rigaku Corporation, 3-9-12 Matsubara-Cho, Akishima 196-8666, Japan*

Supplementary information

Supplementary figure 1

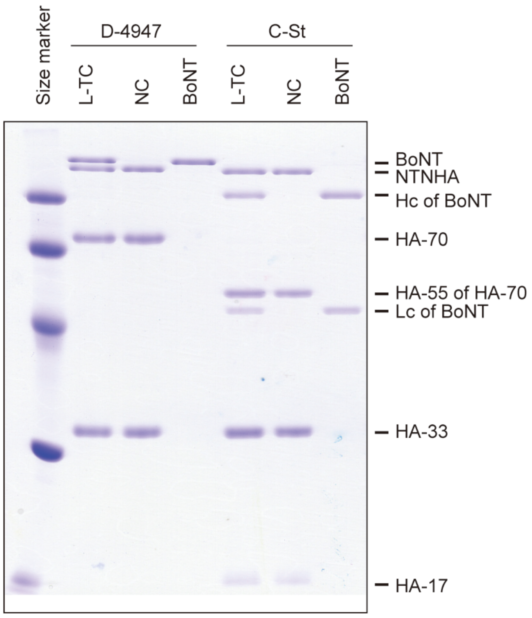

Figure S1. SDS-PAGE banding profiles of the purified L-TC, "Non Toxic" complex and BoNT used in this study.

Supplementary figure 2

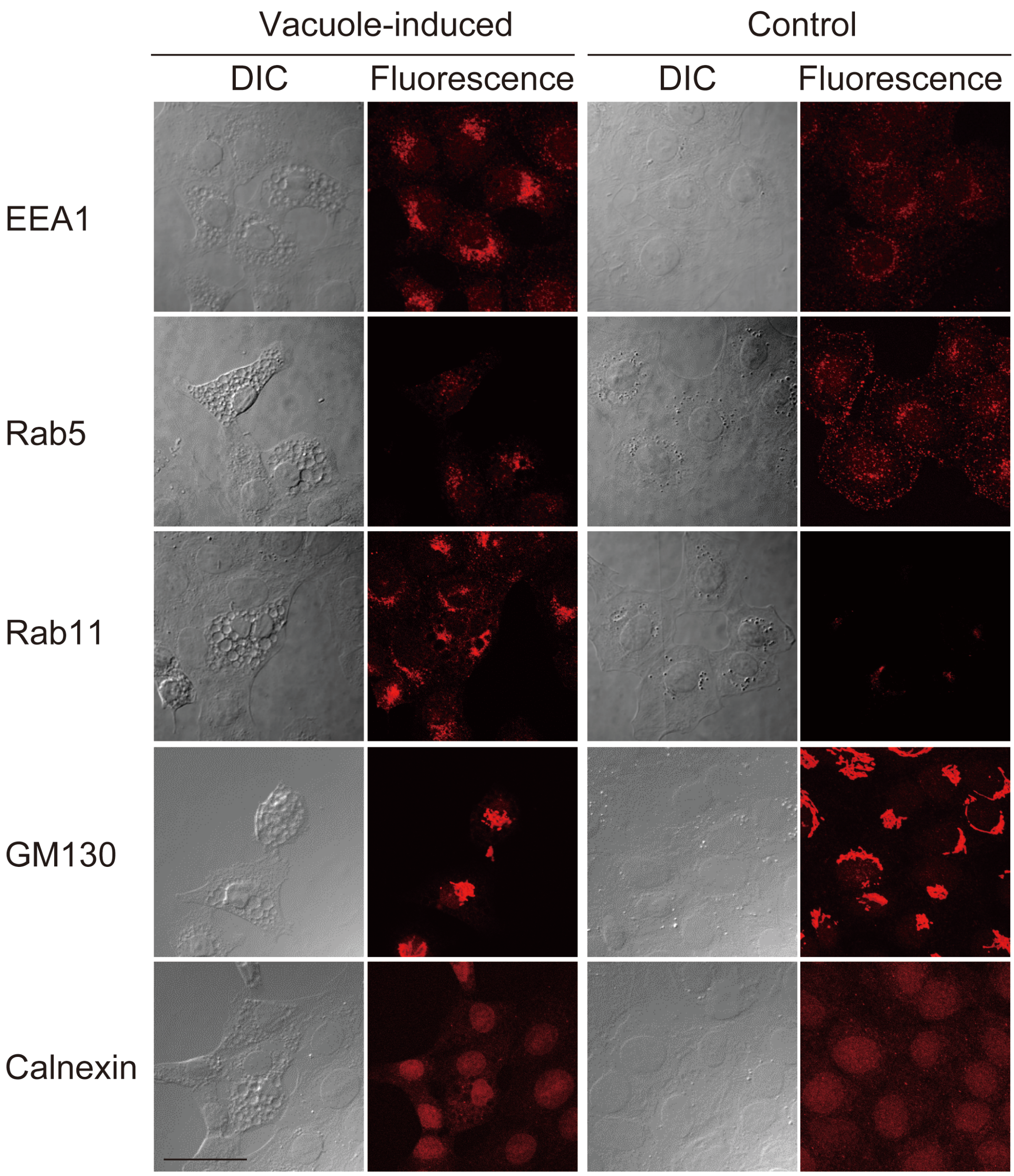

Figure S2. "Non-Toxic" complex-induced vacuoles were not co-localised with the early endosome-specific EEA-1 and Rab5, recycling endosome-specific Rab11, Golgi-specific GM130, and endoplasmic reticulum-specific calnexin. The control cells show the localisation of

each protein in the cell without the exposure to “Non-Toxic” complex. Scale bar: 50 μm.

Movie S1. Solution structure of the L-TC, resembling a bird spreading it wings, revealed by small-angled X-ray scattering (SAXS). Crystal structure of serotype A BoNT/NTNHA complex (red for BoNT and light green for NTNHA), serotype C HA-70 trimer (yellow), and serotype D HA-33/HA-17 trimer (blue for HA-33 and light blue for HA-17) were docked into the SAXS dummy residual model.
